# p53 inhibits OTUD5 transcription to promote GPX4 degradation and induce ferroptosis in gastric cancer

**DOI:** 10.1002/ctm2.70271

**Published:** 2025-03-11

**Authors:** Junjing Zhang, Tongguan Tian, Xinxing Li, Kai Xu, Yao Lu, Xia Li, Xinyu Zhao, Ziyi Cui, Zhenxiang Wang, Yuefan Zhou, Yixin Xu, Hongchen Li, Yan Zhang, Yu Du, Lei Lv, Yanping Xu

**Affiliations:** ^1^ Department of Gastrointestinal Surgery Tongji Hospital, Shanghai Key Laboratory of Signaling and Disease Research, Frontier Science Center for Stem Cell Research, School of Life Sciences and Technology, Tongji University Shanghai China; ^2^ International & Talent Office Tongji Hospital, Tongji University Shanghai China; ^3^ Department of Biochemistry and Molecular Biology, MOE Key Laboratory of Metabolism and Molecular Medicine, School of Basic Medical Sciences Fudan University Shanghai China

**Keywords:** deubiquitinase, ferroptosis, GPX4, OTUD5, p53

## Abstract

**Background:**

Gastric cancer is one of the most prevalent malignant tumors within the digestive system, and ferroptosis playing a crucial role in its progression. Glutathione peroxidase 4 (GPX4), a key negative regulator of ferroptosis, is highly expressed in gastric cancer and contributes to tumor growth. Targeting the regulation of GPX4 has emerged as a promising approach to induce ferroptosis and develop effective therapy for gastric cancer.

**Methods:**

To confirm that OTUD5 is a deubiquitinase of GPX4 and regulates ferroptosis, we performed Western blotting, Co‐IP, immunofluorescence, quantitative real‐time PCR, Ub assay and flow cytometry experiments. To explore the physiological function of OUTD5, we knocked out the Otud5 gene in the mouse gastric cancer cell line (MFC) using CRISPR‐Cas9 and eatablished the subcutaneous tumour model. Immunohistochemistry (IHC) analysis was used to inveatigate the pathological correlation in human gastric cancer.

**Results:**

We report that ovarian tumor domain‐containing 5 (OTUD5) interacts with, deubiquitylates and stabilizes GPX4. OTUD5 depletion destabilizes GPX4, promotes lipid peroxidation and sensitizes gastric cancer cells to ferroptosis. Moreover, the p53 activator nutlin‐3a suppresses OTUD5 transcription, leading to GPX4 degradation and ferroptosis of gastric cancer cells. Notably, only wild‐type p53 has the capacity to inhibit OTUD5 transcription, while p53 mutations or deficiencies correlate with increased OTUD5 expression, promoting gastric cancer progression. Additionally, OTUD5 silencing and nutlin‐3a‐induced GPX4 degradation enhances the sensitivity of gastric cancer cells to ferroptosis in vivo. Subsequently, the p53/OTUD5/GPX4 axis is confirmed in clinical gastric cancer samples.

**Conclusion:**

Collectively, these findings elucidate a mechanism whereby p53 inactivation upregulates OTUD5 transcription to deubiquitylate and stablize GPX4, resulting in ferroptosis inhibition and gastric cancer progression. This discovery highlights the potential therapeutic value of targeting OTUD5 to promote ferroptosis in p53‐inactivated gastric cancer.

**Key points:**

- OTUD5 mediates GPX4 deubiquitination to regulate its stability.
- Deletion of OTUD5 promotes ferroptosis and inhibits tumor growth.
- Wild type p53 inhibits OTUD5 transcription, thereby promoting GPX4 degradation and inhibiting the development of gastric cancer.
- OTUD5, GPX4 expression and p53 activity are highly correlated and correlates with clinical progression in STAD.

## Graphical abstract

In gastric cancer cells harbouring p53 mutations, ovarian tumour domain‐containing 5 (OTUD5) transcription is upregulated due to the inactivation of p53, allowing OTUD5 to stabilise glutathione peroxidase 4 (GPX4) protein, inhibit ferroptosis and thereby promoting gastric cancer progression

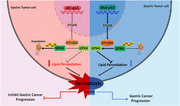

## 1 INTRODUCTION

Recently, the emerging concept of ferroptosis, an iron‐dependent form of cell death, has attracted attention as a potential avenue for cancer therapies.^1^, ^2^ Ferroptosis is primarily characterised by its dependence on iron ions and the generation of lipid peroxides (LPOs), and is distinct from other programmed cell death pathways such as apoptosis, necroptosis and autophagy.^3^, ^4^ Due to the high sensitivity of tumour cells to iron metabolism, ferroptosis has attracted considerable attention in recent years.^5^ Glutathione peroxidase 4 (GPX4), a selenium‐dependent protein, presents in three forms in mammals (mitochondrial subtype mGPX4, nuclear subtype nGPX4 and cytoplasmic subtype cGPX4), wherein only the cytoplasmic subtype cGPX4 is involved in ferroptosis and embryonic development.^6^ By consuming glutathione, cytoplasmic GPX4 can decrease the formation of polyunsaturated LPOs and protect cells from ferroptosis.^7^ Inhibition of GPX4 with erastin or RSL3 would result in extensive lipid peroxidation, thereby facilitating ferroptosis.^8^ However, direct drugs targeting of GPX4 leads to significant drug toxicity, impeding the drug development.^9^ Therefore, targeting the regulation of GPX4, especially post‐translational regulation, has emerged as a new strategy to induce ferroptosis in cancer cells.^10^, ^11^

Protein ubiquitination is one of the most important post‐translational modifications of proteins. Ubiquitination modification primarily takes place in the late stage of protein production, participating in the regulation of DNA damage repair,^12^ tumour development,^13^ immunity^14^ and so on. Ubiquitination is regulated by ubiquitin activating enzyme, ubiquitin conjugating enzyme and ubiquitin ligase (E1–E2–E3) to promote protein degradation via proteasome,^15^, ^16^ while the deubiquitinases (DUBs) can cleave the ubiquitin chain and stabilise the proteins. The regulation of ferroptosis in tumour cells by the ubiquitin system has received growing attentions. Ovarian tumour domain‐containing 5 (OTUD5), recognised as a DUB, plays different roles in different types of cancer.^17^ In bladder, breast and cervical cancers, OTUD5 exhibits pro‐carcinogenic effects, whereas in lung and liver cancers, it plays an anti‐carcinogenic role. This diversity is attributed to the distinct target proteins of OTUD5 within various cancer types, leading to divergent impacts on tumourigenesis. However, whether OTUD5 regulates ferroptosis in gastric cancer remains poorly understood.

The *p53* gene is pivotal in regulating cellular proliferation, apoptosis and metabolism.^18^ Recent research suggests that p53 can influence cellular oxidative stress responses by directly or indirectly modulating the expression of multiple genes.^19^, ^20^ It was reported that p53 transcriptionally suppresses SLC7A11, which diminishes glutathione synthesis and promotes ferroptosis.^21^ p53 can also induce ferroptosis in tumour cells through the GPX4‐independent enzyme ALOX12.^22^ Furthermore, p53 has been shown to upregulate the expression of YAP1 and SAT1, enhance ACSL4 expression and augment ALOX15 enzyme activity, thereby increasing the production of LPOs.^23^, ^24^ However, the exact mechanisms by which p53 modulates the role of GPX4 in iron metabolism and ferroptosis are not fully understood.

In this study, we aim to delineate the regulatory network among p53, OTUD5 and GPX4, and to ascertain their contributions to ferroptosis in gastric cancer. Our research may reveal novel therapeutic targets and facilitate the development of personalised interventions for gastric cancer. Specifically, the interaction among p53, OTUD5 and GPX4 underscores the susceptibility of gastric cancer cells to ferroptosis and the necessity of innovative therapeutic approaches. A comprehensive understanding of these mechanisms is important for advancing our knowledge of the intricate regulatory processes that govern ferroptosis, potentially leading to the development of more targeted and effective therapies for gastric cancer.

## 2 RESULTS

### 2.1 OTUD5 interacts with GPX4

**FIGURE 1:**
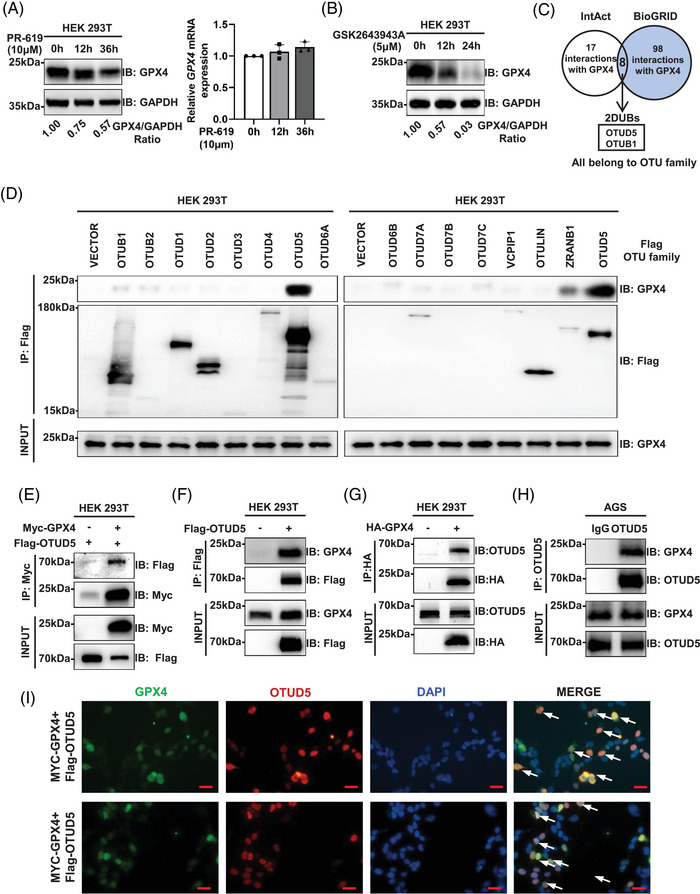
Ovarian tumour domain‐containing 5 (OTUD5) interacts with glutathione peroxidase 4 (GPX4). (A, B) Western blot analysis of GPX4 levels in human embryonic kidney 293 cell line 293T (HEK293T) treated with 10 µM PR‐619 (A) or 5 µM GSK2643943A (B) for different times as indicated. Comparison of GPX4 mRNA levels in HEK293T treated with 10 µM PR‐619 for different time as indicated. mRNA was analysed by quantitative polymerase chain reaction (qPCR), *n* = 3. (C) The Venn diagram illustrates the overlap of GPX4 protein–protein interaction data from IntAct and BioGRID databases. (D) Screening of putative GPX4‐interacting deubiquitinases (DUBs) using 13 plasmids encoding different DUBs of the OTU family. Plasmids encoding Flag‐tagged OTUs were transfected into HEK293T cells for 24 h, cells were harvested for co‐immunoprecipitation (co‐IP) with Flag beads followed by western blot analysis with GPX4 antibodies. (E) Ectopically expressed GPX4 binds to OTUD5. HEK293T cells were transiently transfected with Myc‐GPX4 and Flag‐OTUD5. Cell lysates were subjected to co‐IP with a Myc antibody, analysed by western blot. (F, G) Co‐IP followed by western analysis of the interaction between GPX4 and OTUD5 in HEK293T cells. Plasmids encoding Flag‐OTUD5 (F) or HA‐GPX4 (G) were transfected into HEK293T cells, which were subjected to co‐IP experiments with beads coated with antibodies recognising Flag or HA, followed by western blot analysis. (H) Co‐IP followed by western analysis of the interaction between endogenous GPX4 and OTUD5 in human gastric adenocarcinoma (AGS) cells. (I) Staining of GPX4 (green), OTUD5 (red) and DAPI (blue) in HEK293T cells. Scale bars represent 20 µm.

Previous studies have indicated that GPX4 can be degraded by the ubiquitin‐proteasome pathway.^25^, ^26^ To determine whether GPX4 is regulated by DUB, we treated the cells with DUB inhibitors (PR‐619 and GSK2643943A) and observed a time‐dependent degradation of GPX4 protein levels without a corresponding change in GPX4 mRNA levels (Figure 1A,B). These suggest that DUBs can modulate GPX4 protein level independently of transcriptional level.

To identify the DUB regulating GPX4, we utilised the public protein interaction databases, IntAct (http://www.ebi.ac.uk/intact) and BioGRID (http://www.thebiogrid.org), to search for potential proteins interacting with GPX4 and identified two DUBs, OTUD5 and OTUB1, both are members of the OTU family of DUBs (Figure 1C). Next, we conducted co‐immunoprecipitation (co‐IP) experiments to determine which OTU family member interacts with GPX4. The results demonstrated that OTUD5 exhibited the strongest interaction with GPX4 in human embryonic kidney 293 cell line 293T (HEK293T) cells (Figure 1D).

Next, we confirmed the interaction between OTUD5 and GPX4. Co‐IP showed that OTUD5 interacts with GPX4 in cells (Figure 1E–H), which was further confirmed by their spatial proximity through immunofluorescence (Figure 1I).

### 2.2 OTUD5 deubiquitinates and stabilises GPX4

**FIGURE 2:**
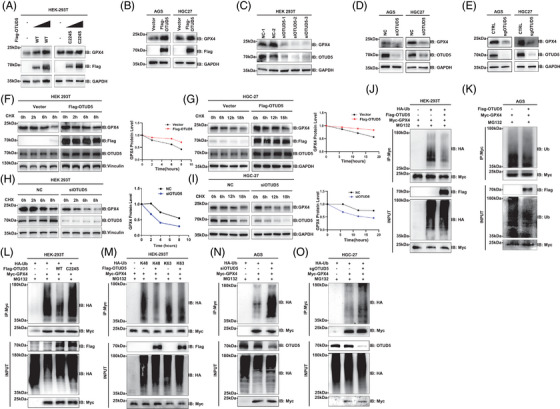
Ovarian tumour domain‐containing 5 (OTUD5) deubiquitinates and stabilises glutathione peroxidase 4 (GPX4). (A) Western blot analysis of endogenous GPX4 levels in human embryonic kidney 293 cell line 293T (HEK293T) cells transfected with increasing amounts of Flag‐OTUD5 wild type or C224S catalytic mutant as indicated. (B) Western blot analysis of endogenous GPX4 levels in human gastric adenocarcinoma (AGS) and human gastric cancer 27 (HGC‐27) human gastric cancer cells transfected with Flag‐OTUD5. (C) Western blot analysis of endogenous GPX4 levels in HEK293T cells transfected with siNC or si*OTUD5* as indicated. (D) Western blot analysis of endogenous GPX4 levels in AGS and HGC‐27 cells transfected with siNC or si*OTUD5*. (E) Western blot analysis of endogenous GPX4 levels in AGS and HGC‐27 cells with *OTUD5* knocked out. (F, G) Half‐life analysis of GPX4 in HEK293T (F) and HGC‐27 (G) cells transfected with Flag‐OTUD5 and treated with 200 µg/mL cycloheximide (CHX) for different times as indicated. GPX4 protein levels were quantified using ImageJ software. (H, I) Half‐life analysis of GPX4 in HEK293T (H) and HGC‐27 (I) cells transfected with si*OTUD5* and treated with 200 µg/mL CHX for different times as indicated. GPX4 protein levels were quantified using ImageJ software. (J) Western blot analysis of GPX4 ubiquitination levels in HEK293T cells transfected with Myc‐GPX4, Flag‐OTUD5 and HA‐Ub for 24 h and followed by MG132 (20 µM) treatment for 6 h. GPX4 was immunoprecipitated with Myc beads and ubiquitination was detected with HA antibody. (K) Western blot analysis of GPX4 ubiquitination levels in AGS cells transfected with Myc‐GPX4 and Flag‐OTUD5 as indicated for 24 h and followed by MG132 (20 µM) treatment for 6 h. Ubiquitinated GPX4 proteins were determined with ubiquitin antibody. (L) Western blot analysis of GPX4 ubiquitination levels in HEK293T cells transfected with Myc‐GPX4, Flag‐OTUD5 or Flag‐OTUD5‐C224S and HA‐Ub as indicated for 24 h and treated with MG132 (20 µM) for 6 h. GPX4 was immunoprecipitated with Myc beads and ubiquitination was blotted with HA antibody. (M) Western blot analysis of GPX4 ubiquitination in HEK293T cells transfected with indicated plasmids for 24 h and treated with MG132 (20 µM) for 6 h. GPX4 was immunoprecipitated with Myc beads and ubiquitination was detected with HA antibody. (N) Western blot analysis of GPX4 ubiquitination in AGS cells transfected with Myc‐GPX4 and HA‐Ub for 24 h after si*OTUD5* transfection for 48 h and then treated with MG132 (20 µM) for 6 h. GPX4 was immunoprecipitated with Myc beads and ubiquitination was detected with HA antibody. (O) Western blot analysis of GPX4 ubiquitination levels in control and *OTUD5* knockout HGC‐27 cells transfected with Myc‐GPX4 and HA‐Ub as indicated.

To determine whether OTUD5 regulates GPX4 expression, HEK293T cells were transfected with either wild‐type OTUD5 or a mutant variant (C224S‐OTUD5). Results revealed that overexpression of wild‐type OTUD5 led to elevated GPX4 protein levels, whereas the catalytic mutant C224S‐OTUD5 did not influence GPX4 expression (Figure 2A), indicating that OTUD5 upregulated GPX4 protein levels depending on its DUB activity. Similar results were observed in both human gastric adenocarcinoma (AGS) and human gastric cancer 27 (HGC‐27) cells (Figure 2B). Conversely, knockdown or knockout of *OTUD5* decreased the endogenous GPX4 protein levels in HEK293T, AGS and HGC‐27 cells (Figure 2C–E). Notably, OTUD5 overexpression, knockdown or knockout did not impact the mRNA levels of GPX4 (Figure S1A–C), indicating that OTUD5 upregulates GPX4 expression independently of transcriptional level in gastric cancer cells.

Subsequently, we determined the regulation of OTUD5 on the half‐life of GPX4. The results demonstrated that OTUD5 overexpression extended the half‐life of GPX4 (Figure 2F,G). Conversely, knockdown of OTUD5 shortened the half‐life of GPX4 in both HEK293T and HGC‐27 cells (Figure 2H,I). Of note, the half‐life of GPX4 is relatively long, which may be attributed to the essential role of GPX4, as its complete ablation is embryonically lethal. Therefore, identifying proteins that regulate GPX4 degradation is imperative for promoting ferroptosis without leading to the total elimination of GPX4.

Next, we examined whether OTUD5 mediates the deubiquitination of GPX4 and found that overexpression of OTUD5 led to a significant reduction of GPX4 polyubiquitination (Figure 2J,K). Notably, the expression of wild‐type OTUD5 (OTUD5‐WT), but not the catalytically inactive mutant (OTUD5‐C224S) reduced GPX4 ubiquitination (Figure 2L). These data suggested that OTUD5 deubiquitinates GPX4, thereby enhancing its protein stability. Furthermore, OTUD5 significantly reduced K48‐linked ubiquitination, but had no effect on the K63‐linked ubiquitination of GPX4 (Figure 2M), indicating that OTUD5 specifically targets K48‐linked ubiquitination of GPX4. Additionally, we found that knockdown or depletion of *OTUD5* markedly increased GPX4 ubiquitination (Figure 2N,O). In conclusion, these results demonstrated that OTUD5 mediates the K48‐linked deubiquitination of GPX4 and stabilises GPX4.

### 2.3 OTUD5 suppresses ferroptosis via GPX4

**FIGURE 3:**
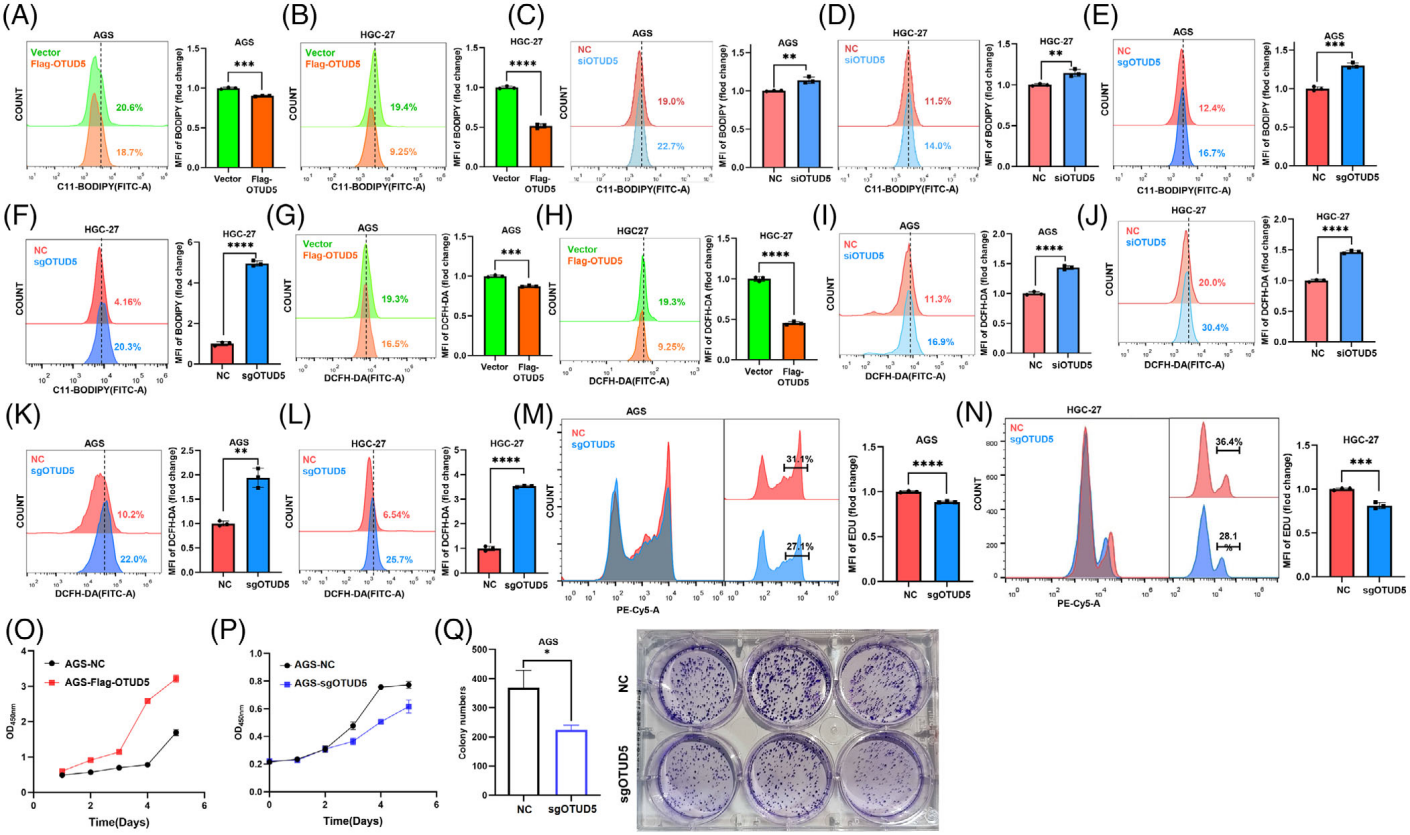
Ovarian tumour domain‐containing 5 (OTUD5) inhibits ferroptosis of gastric cancer cells via glutathione peroxidase 4 (GPX4). (A, B) Lipid peroxidation levels were determined in human gastric adenocarcinoma (AGS) (A) and human gastric cancer 27 (HGC‐27) (B) cells transfected with vector or Flag‐OTUD5 as indicated using C11‐BODIPY staining. (C, D) Lipid peroxidation levels were determined in AGS (C) and HGC‐27 (D) cells transfected with NC or si*OTUD5* as indicated using C11‐BODIPY staining. (E, F) Lipid peroxidation levels were determined in control and *OTUD5* knockout AGS (E) and HGC‐27 (F) cells using C11‐BODIPY staining. (G, H) Reactive oxygen species (ROS) levels were determined in AGS (G) and HGC‐27 (H) cells transfected with vector or Flag‐OTUD5 as indicated using DCFH‐DA staining. (I, J) ROS levels were determined in AGS (I) and HGC‐27 (J) cells transfected with NC or si*OTUD5* as indicated using DCFH‐DA staining. (K, L) ROS levels were determined in control and *OTUD5* knockout AGS (K) and HGC‐27 (L) cells using DCFH‐DA staining. (M, N) The proliferation of control and *OTUD5* knockout AGS (M) and HGC‐27 (N) cells was determined through flow cytometry following EdU staining. (O) The proliferation of control and OTUD5 overexpressed AGS cells was determined through CCK‐8 assay for 5 days. (P) The proliferation of control and *OTUD5* knockout AGS cells was determined through CCK‐8 assay for 5 days. (Q) The clone formation ability of control and *OTUD5* knockout AGS cells was determined. Data are shown as the mean ± SD (*n* = 3).

Given the critical role of GPX4 in the process of ferroptosis, we extended our investigation to the impact of OTUD5 on ferroptosis in gastric cancer cells. We found that overexpression of OTUD5 led to a reduction in intracellular lipid peroxidation in both AGS and HGC‐27 cells (Figure 3A,B). Conversely, knockdown or knockout of *OTUD5* resulted in elevated levels of lipid peroxidation in these cells (Figure 3C–F), indicating an important role of OTUD5 in modulating lipid peroxidation. To further determine whether OTUD5 regulates cellular lipid peroxidation through GPX4, we knocked down GPX4 in cells overexpressing OTUD5. The results showed that even in the context of OTUD5 overexpression, GPX4 knockdown still resulted in a significant increase of cellular lipid peroxidation levels (Figure S2A). In contrast, overexpression of GPX4 can reverse OTUD5 knockdown‐induced cellular lipid peroxidation (Figure S2B). These results suggested that the regulation of cellular lipid peroxidation levels by OTUD5 is dependent on the expression and function of GPX4. Next, we assessed the effects of OTUD5 on reactive oxygen species (ROS) levels, which indicate cellular redox status, and found that the results were consistent with those of lipid peroxidation (Figure 3G–L). These data suggested that OTUD5 might regulate the sensitivity of AGS cells to ferroptosis. To test this hypothesis, we examined the effects of two ferroptosis inducers, erastin and RSL3, in OTUD5 overexpression or knockdown AGS cells. The results showed that OTUD5 overexpression inhibited RSL3 and erastin‐induced lipid peroxidation (Figure S2C), whereas OTUD5 knockdown enhanced this effect (Figure S2D). In addition, we examined the effects of RSL3 and erastin on OTUD5 protein levels and its activity to GPX4 deubiquitination, and found that the two compounds affected neither OTUD5 protein levels nor its deubiquitinating activity (Figure S2E,F). Collectively, these results imply that OTUD5 orchestrates ferroptosis in gastric cancer cells.

Moreover, we evaluated the impact of *OTUD5* knockout on the cell cycle of gastric cancer cells by an EdU incorporation assay, and the results revealed that OTUD5 depletion decreased the proportion of cells in S phase, indicating impaired cell proliferation (Figure 3M,N). Consistently, overexpression of OTUD5 promoted the proliferation, while OTUD5 depletion suppressed the proliferation of gastric cancer cells (Figure 3O,P). Furthermore, colony formation assays demonstrated the reduced proliferative capacity of gastric cancer cells upon OTUD5 depletion (Figure 3Q). These data collectively support the notion that OTUD5 inhibits the proliferation of gastric cancer cells.

### 2.4 Wild‐type p53 transcriptionally suppresses OTUD5 to promote ferroptosis in gastric cancer cells

**FIGURE 4:**
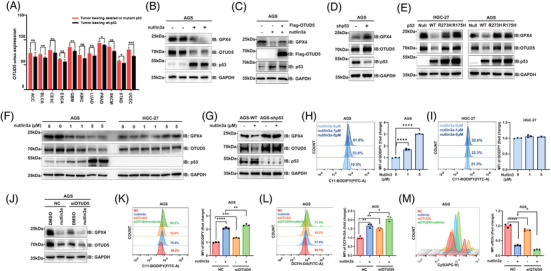
Wild‐type p53 transcriptionally suppresses ovarian tumour domain‐containing 5 (OTUD5) to promote ferroptosis in gastric cancer cells. (A) The mRNA expression levels of OTUD5 in different types of human tumours harbouring wild‐type or mutant/deletion p53 as indicated (data collected from http://tcga‐data.nci.nih.gov/tcga). (B) Western blot analysis of glutathione peroxidase 4 (GPX4), OTUD5 and p53 level in human gastric adenocarcinoma (AGS) treated with 5 µM nutlin3a for 24 h. (C) Western blot analysis of GPX4 levels in AGS cells overexpressing OTUD5 treated with 5 µM nutlin3a for 24 h. (D) Protein levels of GPX4, OTUD5 and p53 were determined by western blot in shCTRL and sh*p53* AGS cells. (E) Protein levels of GPX4 and OTUD5 were determined by western blot in AGS and human gastric cancer 27 (HGC‐27) cells expressing p53 wild type or mutants (R175H, R273H) as indicated. R, arginine; H, histidine. (F, G) Western blot analysis of GPX4, OTUD5 and p53 level in AGS, HGC‐27 (F) and AGS p53‐knockdown (G) cells stimulated with nutlin3a as indicated for 24 h. (H, I) Lipid peroxidation levels in AGS (H) and HGC‐27 (I) cells were examined using C11‐BODIPY staining stimulated with different concentrations of nutlin3a as indicated for 24 h. (J) Western blot analysis of GPX4 and OTUD5 levels in CTRL and si*OTUD*5 AGS cells treated with 5 µM nutlin3a for 24 h. (K) Lipid peroxidation levels were determined in CTRL and si*OTUD5* cells treated with 5 µM nutlin3a for 24 h in AGS cells. (L) Reactive oxygen species (ROS) levels in AGS cells were examined in CTRL and si*OTUD5* cells treated with 5 µM nutlin3a for 24 h in AGS cells by DCFH‐DA staining. (M) The proliferation of CTRL and si*OTUD5* AGS cells treated with 5 µM nutlin3a as indicated was determined through flow cytometry following EdU staining.

Previous studies have suggested that p53 can promote ferroptosis, however, whether OTUD5–GPX4 axis plays a role in this regulatory process remains unclear. The Cancer Genome Atlas (TCGA) analysis showed that the mRNA level of OTUD5 was frequently higher in multiple tumours harbouring a p53 mutation than that observed in p53 wild‐type tumours, including stomach adenocarcinoma (STAD; Figure 4A). Moreover, treatment of AGS gastric cancer cells with nutlin‐3a, a known p53 activator, led to a pronounced reduction in the protein levels of OTUD5 and GPX4 (Figure 4B), as well as an inhibition of cell proliferation (Figure S3A). Notably, the mRNA level of GPX4 remained unchanged upon nutlin‐3a treatment (Figure S3B), indicating that p53 might regulate the protein stability of GPX4. Therefore, we examined the effect of nutlin‐3a on OTUD5 and the E3 ubiquitin ligases (TRIM26,^27^ MARCHF1,^28^ STUB1^25^ and RC3H1^29^) reported to regulate GPX4. The results showed that nutlin‐3a significantly reduced the mRNA level of OTUD5, while it had no effect on the transcription levels of the E3 ubiquitin ligases (Figure S3C). These data demonstrated that p53 might regulate GPX4 stability via OTUD5. In line with this, overexpression of OTUD5 can block p53 activation‐induced GPX4 downregulation (Figure 4C). Moreover, knocking down *p53* in AGS cells increased OTUD5 and GPX4 levels (Figure 4D). Consistently, ectopic expression of wild‐type, but not mutant p53, downregulated GPX4 and OTUD5 levels in HGC‐27 and AGS cells (Figure 4E). These observations imply that p53 is a negative regulator of OTUD5 transcription, resulting in reduced GPX4 protein levels in gastric cancer cells.

Interestingly, the expression levels of OTUD5 and GPX4 decreased progressively with the increasing concentrations of nutlin‐3a in AGS cells, while there was no significant alteration in the expression levels of OTUD5 and GPX4 following nutlin‐3a treatment in HGC‐27 cells or p53‐knockdown AGS cells (Figures 4F,G and S3D–G). The p53 in HGC‐27 cells is mutated^30^ and undetectable (Figure S3H). Therefore, nutlin‐3a cannot regulate OTUD5 and GPX4 in HGC‐27 cells (Figure 4F). Consistently, nutlin‐3a increased the lipid peroxidation levels in a dose‐dependent manner in AGS cells (Figure 4H), while in HGC‐27 cells, nutlin‐3a had no effect on lipid peroxidation levels (Figure 4I).

Subsequently, we treated control and OTUD5 knockdown cells with nutlin‐3a. Results showed that OTUD5 knockdown impaired the effect of nutlin‐3a on GPX4, and a decrease in the protein levels of OTUD5 and GPX4 was observed, with the most pronounced reduction occurring under the combination treatment of si*OTUD5* and nutlin‐3a (Figure 4J). Consistent results were obtained when assessing lipid peroxidation levels (Figure 4K), cellular ROS (Figure 4L) and cell proliferation (Figure 4M). These findings demonstrate that both OTUD5 knockdown and nutlin‐3a treatment can induce ferroptosis and suppress cell proliferation. To determine whether p53 directly regulates OTUD5, ChIP‐qPCR experiment was performed and results showed that p53 could bind to the OTUD5 promoter region (Figure S3I). Collectively, these results reveal the role of p53–OTUD5–GPX4 axis in ferroptosis of gastric cancer cells.

### 2.5 OTUD5 suppresses ferroptosis and promotes tumour growth in vivo

**FIGURE 5:**
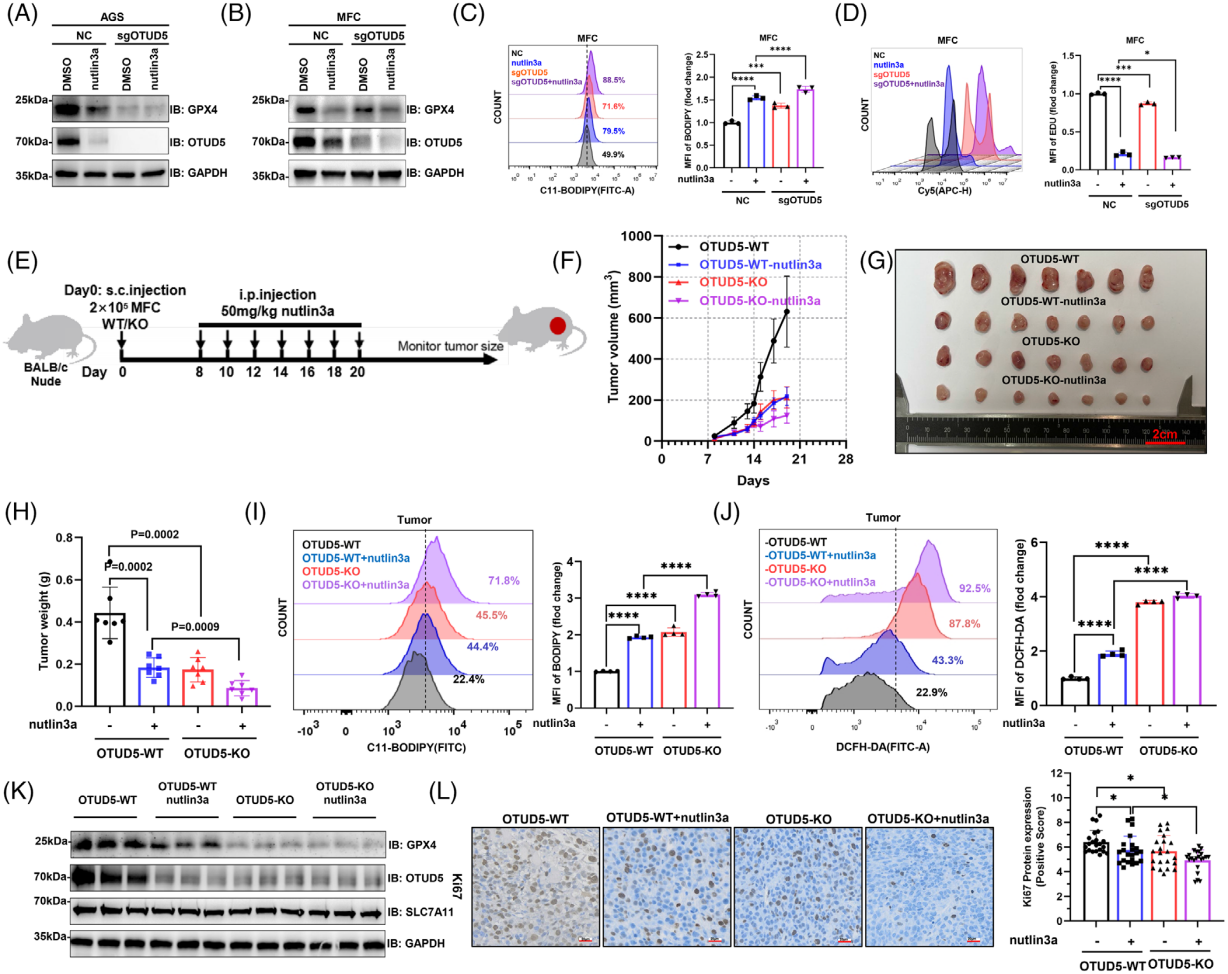
Ovarian tumour domain‐containing 5 (OTUD5) suppresses ferroptosis and promotes tumour growth in vivo. (A, B) Western blot analysis of glutathione peroxidase 4 (GPX4) and OTUD5 levels in CTRL and sg*OTUD5* human gastric adenocarcinoma (AGS) (A) and mouse forestomach cancer (MFC) (B) cells treated with 5 µM nutlin3a for 24 h. (C) Lipid peroxidation levels were determined in CTRL and sg*OTUD5* MFC cells treated with 5 µM nutlin3a for 24 h. (D) The proliferation of CTRL and sg*OTUD5* MFC cells treated with 5 µM nutlin3a was determined through flow cytometry following EdU staining. (E–H) Tumour volume was determined and analysed within 2 weeks for OTUD5‐WT, OTUD5‐WT + nutlin3a, OTUD5‐KO, OTUD5‐KO + nutlin3a. A total of 2 × 10^5^ WT or OTUD5‐KO MFC cells were injected into nude mice and treated with nutlin3a as indicated (E). Tumour volume (F, G) and tumour weight (H) were determined and analysed. Data represent mean ± SEM for 7 tumours. (I) Lipid peroxidation levels in tumour tissues were examined using C11‐BODIPY staining. (J) Reactive oxygen species (ROS) levels in tumour tissues were examined using C11‐BODIPY staining. (K) Western blot analysis of GPX4, OTUD5, SLC7A11 protein levels in MFC tumour tissues. (L) Representative images of IHC staining of Ki67 in MFC‐derived tumours tissues (scale bars, 20 µm). Images were quantified (right panel) by ImageJ. Data represent mean ± SD of six biological replicates and two technical replicates with *p* value was determined by Student's *t*‐test.

Next, we employed CRISPR‐Cas9 genome editing technology to delete *OTUD5* in AGS and mouse forestomach cancer (MFC) cells, and found that depletion of *OTUD5* significantly suppressed the expression of GPX4 in both of the cells (Figure 5A,B). We treated control and *OTUD5*‐KO cells with nutlin‐3a and observed that knockout of *OTUD5*, in combination with nutlin‐3a treatment, led to a reduction in protein levels of OTUD5 and GPX4 (Figure 5A,B), thereby triggering ferroptosis in cancer cells and consequently inhibiting cell proliferation (Figure 5C,D).

Next, we examined the regulatory role of OTUD5 on gastric tumour growth using a mouse xenograft model (Figure 5E). Results showed that *OTUD5*‐KO significantly suppressed tumour growth in vivo (Figure 5F–H). Notably, the inhibitory effect of *OTUD5*‐KO on tumour growth was found to be comparable to that of nutlin‐3a treatment, with the combination of both treatments yielding the most pronounced inhibitory effect (Figure 5F–H). Consistently, both *OTUD5*‐KO and nutlin‐3a treatment significantly increased the levels of lipid peroxidation (Figure 5I) and ROS (Figure 5J) in mouse tumours.

Moreover, we examined OTUD5 and GPX4 protein levels in different groups of MFC tumour tissues collected. The result showed that the expression levels of OTUD5 and GPX4 were significantly reduced in both nutlin3a‐treated and *OTUD5* KO groups compared to the control group (Figure 5K). In addition, analysis of Ki67 staining of tumour tissues revealed that *OTUD5*‐KO and nutlin‐3a treatment had similar inhibitory impacts on tumour proliferation, with the combined treatment exhibiting the most significant efficacy (Figure 5L). This implies that p53 influences ferroptosis by repressing OTUD5 in gastric cancer.

### 2.6 OTUD5, GPX4 expression and p53 activity is correlated with clinical features of gastric cancer patients

**FIGURE 6:**
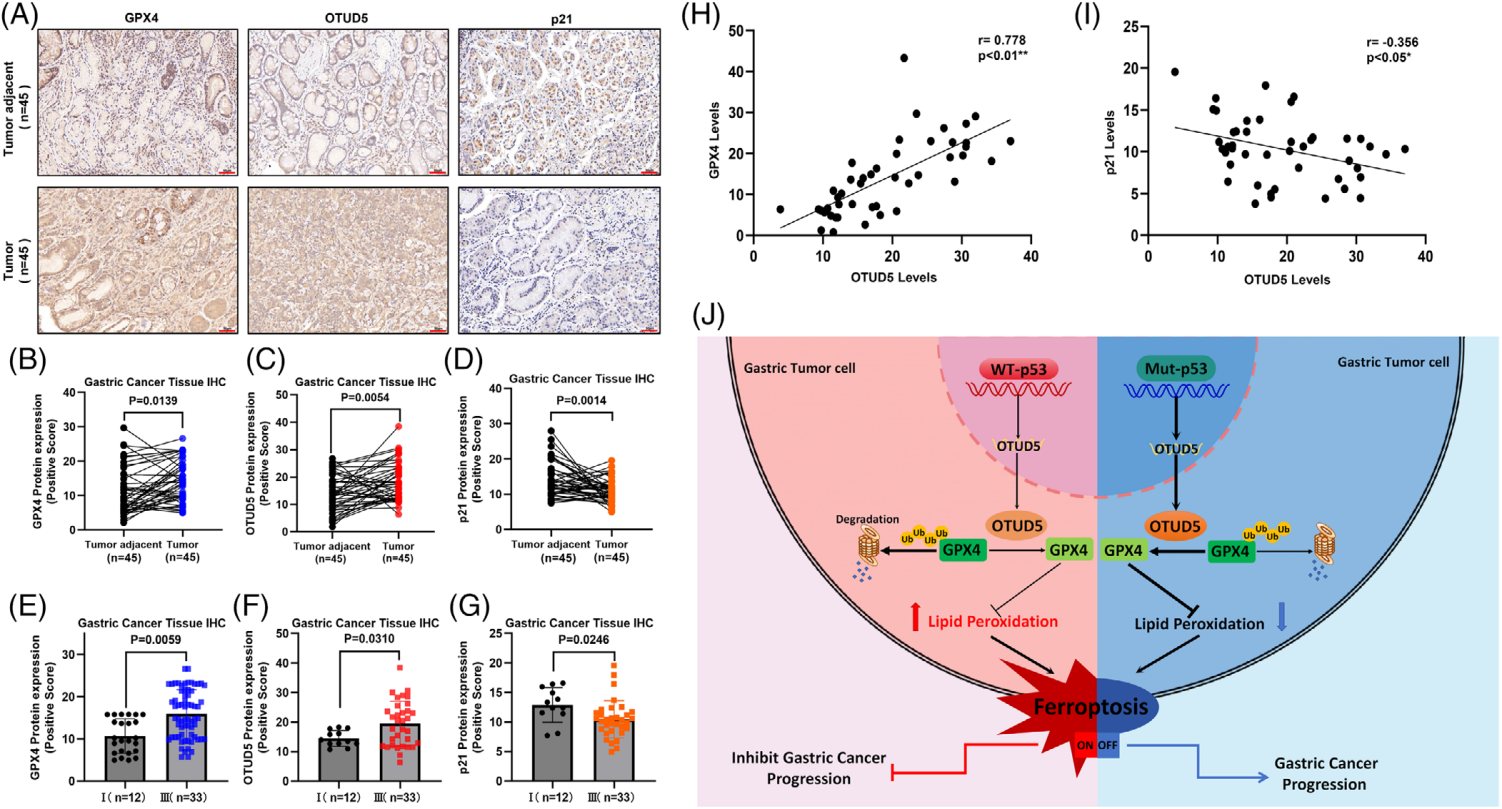
Ovarian tumour domain‐containing 5 (OTUD5), glutathione peroxidase 4 (GPX4) expression and p53 activity is correlated with clinical features of gastric cancer patients. (A) Representative images of IHC staining of GPX4, OTUD5 and p21 in human stomach adenocarcinoma (STAD) specimens. Scale bars represent 50µm. (B–D) The quantifications of GPX4 (B), OTUD5 (C) and p21 (D) are shown below. *n* = 45 biological replicates from 15 donors. For each case, three areas highly infiltrated with inflammatory cells were selected. Data represent mean ± SD for 15 specimens. *p* value was obtained by Student's *t*‐test. (E–G) Correlation between GPX4 (E), OTUD5 (F) and p21 (G) expression and tumour progression were analysed. Data were quantified from (A) with I: *n* = 12, III: *n* = 33. Error bars represent mean ± SD from 12 (I), and 33 (III) biological replicates each from three fields. (H, I) Spearman's correlation analysis of the association between OTUD5 and GPX4 (H) and p21 (I) in STAD. The Spearman correlation coefficient (*R*) and *p* value were determined with Spearman's rank test. (J) Working model for regulation of GPX4 stability by p53‐OTUD5 axis.

We further investigated the pathological correlation between OTUD5 and GPX4 in human gastric cancer. We conducted IHC analysis on clinical gastric cancer patient samples to determine the correlation of OTUD5, GPX4 and p21 in STAD, where p21 was used as an indicator of p53 activity. The results showed that the expression levels of OTUD5 and GPX4 were significantly elevated, while the p21 expression decreased in STAD compared to adjacent tissues (Figure 6A–D). More importantly, quantitative analysis revealed a significant positive correlation between OTUD5 and GPX4 protein levels and advanced stages of gastric cancer, while the p21 protein levels showed a negative correlation with advanced stages (Figure 6E–G). Furthermore, GPX4 protein levels were positively correlated with OTUD5 expression levels, while the levels of p21 were negatively correlated to OTUD5 expression levels (Figure 6H,I). Taken together, these results provide clinical evidence for p53‐OTUD5 axis‐mediated regulation of GPX4 protein stability, suggesting that the expression level of OTUD5 is important for ferroptosis of gastric cancer cells.

## 3 DISCUSSION

Gastric cancer is a major global health concern due to its high incidence and mortality rates.^31^ Despite advancements in molecular targeted therapies, the emergence of drug resistance and the elucidation of its underlying mechanisms remain formidable challenges.^32^, ^33^ Therefore, it is crucial to further investigate the pathological mechanisms of gastric cancer and to explore novel therapeutic strategies. Recent research has highlighted the important connection between gastric cancer and ferroptosis, a form of regulated cell death mediated by iron and lipid peroxidation.^1^, ^34^, ^35^

Within the scope of this study, we identified that GPX4, a principal negative regulator of ferroptosis, is markedly overexpressed in gastric cancer patient tissues when compared to normal tissues, suggesting a critical role for GPX4 in enabling gastric cancer cells to evade ferroptosis. Targeting the degradation pathways of GPX4 to induce ferroptosis of gastric cancer cells may present a promising therapeutic approach. However, directly targeting GPX4 with drugs currently causes considerable toxicity, which has hindered the pace of drug development.^10^ Thus, we propose a novel therapeutic strategy and drug development target: the proteins that regulate GPX4 expression. It is known that GPX4 can be degraded via the ubiquitin‐proteasome pathway.^36^ This study reveals that OTUD5, a deubiquitinating enzyme, is a key regulator of GPX4 in gastric cancer cells. Furthermore, we investigated the specific mechanism by which p53 regulates OTUD5 and GPX4 in gastric cancer and found that p53 transcriptionally suppresses OTUD5, thereby regulating ferroptosis in gastric cancer cells. Unlike GPX4, OTUD5 is not indispensable,^37^ and its role varies across different cancers; OTUD5 has been characterised as an oncogene in bladder,^38^ breast^39^ and cervical cancers,^40^ while it exhibits tumour suppressor properties in lung^41^ and liver cancers.^42^ Our data demonstrated that OTUD5 knockout significantly reduced tumour progression (Figure 5E–H). Notably, the anticancer effect of OTUD5 knockout was found to be comparable to that of nutlin‐3a treatment, underscoring the potential of OTUD5 as a therapeutic target for inducing ferroptosis in gastric cancer.

Based on our findings, we propose a working model in which the transcription of OTUD5 is significantly suppressed in p53 wild‐type gastric cancer cells (Figure 4A). This suppression leads to a decrease of OTUD5 mRNA levels, a consequent reduction of GPX4 protein levels, and an increase of LPO accumulation, ultimately resulting in ferroptosis of tumour cells and the inhibition of gastric cancer progression (Figure 6J, left panel). Conversely, in gastric cancer cells harbouring p53 mutations, OTUD5 transcription is upregulated due to the inactivation of p53, allowing OTUD5 to stabilise GPX4 protein, inhibit ferroptosis and thereby promoting gastric cancer progression (Figure 6J, right panel).

Inhibition of OTUD5 holds the potential to induce ferroptosis in gastric cancer, positioning it as a promising target for the development of drug for gastric cancer. Currently, specific inhibitors targeting deubiquitinating enzyme (DUB) have emerged as promising therapeutic agents in drug development. The identification of an inhibitor for OTUD5 could potentially lead to the suppression of GPX4, preventing the onset and progression of gastric cancer.

## 4 MATERIALS AND METHODS

### 4.1 Clinical samples and ethics statement

Tissue samples from patients diagnosed with gastric cancer (STAD) were collected at Tongji Hospital. Informed consent was obtained from all subjects, and the study was ethically approved by Ethics Committee of Tongji Hospital affiliated to Tongji University. The tumours were quickly excised and preserved in a 4% paraformaldehyde (PFA) solution, then embedded in paraffin. This study was carried out in accordance with the ethical principles outlined in the Declaration of Helsinki. The staging of gastric cancer detailed in this manuscript was established by the Clinical Pathology Department of Tongji Hospital, following the diagnostic criteria set forth by the American Joint Committee on Cancer (AJCC).

### 4.2 Subcutaneous tumour model

MFC cells (2 × 10^5^) were injected into the right flank of 5–6‐week‐old male nude mice subcutaneously. Nutlin3a was given via intraperitoneal injection at a dosage of 50 mg/kg every other day over a span of 2 weeks. Tumour size was measured every 2 days by a calliper, with tumour volume calculated using the formula: width^2^ × length × 0 .523.^43^ Mice were euthanised when maximum size of tumour reached 20 mm in diameter and 1500 mm^3^ in volume. Analysis of tumour tissues by immunohistochemistry, western blotting and quantitative polymerase chain reaction (qPCR) according to standard protocols. Additionally, single‐cell suspensions from the tumour tissues were utilised to determine ferroptosis.

### 4.3 Cell culture

AGS, HEK293T, HGC‐27 and MFC cell line were purchased from MeilunBio. These cells were maintained in DMEM/RIPM‐1640 medium (MeilunBio) supplemented with 10% foetal bovine serum (FBS) and 1% penicillin/streptomycin (NCM Biotech#C100C5).

### 4.4 Plasmid construction and cell transfection

The plasmids pCDNA3.1‐Myc‐GPX4 and plvx‐Flag‐OTUD5 were synthesised and constructed with cDNA by Shanghai Tongji Life Sciences Co., Ltd. The plasmids of p53‐WT, p53‐null, p53‐R175H, p53‐R273H, sh‐CTRL and sh‐p53 were kindly provided by the Peng Jiang Research Group at Tsinghua University. Ubiquitin or its mutants (K48 and K63) was inserted into a pCMV backbone with a 4xHA. Cells were transiently transfected with plasmids by EZ‐trans (Life‐iLab).

### 4.5 RNAi

All siRNA oligonucleotides were synthesised in Shanghai QIANKO Biotechnology Company. The siRNA sequences were listed as follows: si‐human‐OTUD5‐1, 5′‐CAGUGG UGAAUCCUAACAA‐3′; si‐human‐OTUD5‐2, 5′‐GACUUUACCACCUACAUUA‐3′; si‐human‐OTUD5‐3, 5′‐GGUGUACCAGUACAGCACA‐3′. Lipofectamine 2000 reagent (Life Technologies) is used for siRNA transfection according to manufacturer's instructions. The efficiency of knockdown was assessed 48–72 h following transfection.

### 4.6 CRISPR‐Cas9

Recombinant lentiviral particles were generated through transient transfection of HEK293T cells following a standard protocol. The sgRNA sequences were listed: human *OTUD5* 1#5′‐CCATTCGTGTTAGCTACCAT‐3′; 2#5′‐CCGATGGTAGCTAACACGAA‐3′; 3#5′‐ACCGGCTAGTCCACTCCTCC‐3′; mouse *Otud5* 1#5′‐ CCATCCGTGTCAGCTACCAC‐3′; 2#5′‐ACTTCACCACCTATATCAAC‐3′; 3#5′‐AGAGATGTACAACCGTCCTG‐3′. For transduction process, viral solutions were incorporated into cell culture medium with polybrene (4 mg/mL), followed by selection with puromycin (10 mg/mL). The efficiency of gene depletion is determined by immunoblotting.

### 4.7 Antibodies and reagents

For western blot, antibodies used are list as follows: anti‐Ub (CST#3936), anti‐K48(CST#8081), anti‐K63 (CST#5621), anti‐OTUD5 (Proteintech, #21002‐1‐AP), anti‐GPX4 (Proteintech, # 67763‐1‐Ig; Hua Bio# ET1706‐45), anti‐p53 (Proteintech, # 60283‐2‐Ig), anti‐GAPDH (Proteintech, # 60004‐1‐Ig), anti‐SLC7A11 (Proteintech, #26864‐1‐AP), anti‐HA (GNI#4510‐HA), anti‐Flag (GNI#4510‐FG), anti‐Myc (GNI#4510‐MC), anti‐HA‐HRP (GNI#4310‐HA), anti‐Flag‐HRP (GNI#4310‐FG), anti‐Myc‐HRP (GNI#4310‐MC), for human GPX4 IHC, anti‐GPX4 (SAB# 32506) was used; for immunofluorescence, Alexa Fluor 488‐labelled goat anti‐mouse IgG (H + L) and Alexa Fluor 555‐labelled donkey anti‐rabbit IgG (H + L) were purchased from Beyotime. For reagents, MG132 (#T2154), NH4Cl (#12125‐02‐9), PR‐619 (#T1862), nutlin3a (#T6023), GSK2643943A (#T11485), RSL3 (#T3646) and erastin (#T1765) were purchased from Target Mol; cycloheximide (CHX, #66‐81‐9) was purchased from Sigma‐Aldrich.

### 4.8 In vivo ubiquitylation assay

HEK293T were transfected with plasmids and/or siRNA for 48 h. Then cells were denatured by boiling for 10  min in SDS buffer (50 mM Tris–HCl, pH 7.5,  0.5 mM EDTA, 1 mM DTT, 1% SDS). Cell lysate was diluted 10 times using an NP‐40 buffer (50 mM Tris pH 7.5, 150 mM NaCl,  0.3% Nonidet P‐40) and determined by IP‐western blotting.

### 4.9 Immunofluorescence

Following 48‐h co‐transfection of FLAG‐OTUD5 and Myc‐GPX4 in HEK293T cells, culture slides underwent washing twice with phosphate‐buffered saline (PBS) and then were fixed in 4% PFA for 15 min at room temperature (RT). Next, cells were blocked with blocking solution at RT for 1 h before incubation with anti‐GPX4 (mouse) and anti‐OTUD5 (Rabbit) at 4°C overnight, secondary antibody Alexa Fluor 488‐labelled Goat Anti‐Mouse IgG (H + L) (Beyotime, A0428) and Alexa Fluor 555‐labelled donkey anti‐rabbit IgG (H + L) (Beyotime, A0453) were used to incubate for another 2 h at RT. After performing 4',6‐diamidino‐2‐phenylindole (DAPI) staining, the slides were treated with antifade reagent and visualised by Olympus microscope (CKX53).

### 4.10 Immunohistochemistry (IHC)

Tumours tissues from subcutaneous MFC mouse tumour model or STAD patients were collected and fixed in 4% PFA solution Tumour samples were subjected to immunostaining with the primary antibodies: OTUD5, GPX4, p21. To quantify the IHC results, immunoreactivity was analysed based on both intensity and area as previously defined.^44^

### 4.11 qRT‐PCR

Total RNA was extracted by the RNA extraction kit (EZB, 15596026CN), and followed by cDNA synthesis by SuperScript II Reverse Transcriptase (Vazyme, R222‐01). For the relative quantification of RNA levels determination, the SYBR Green Master Mix Kit (Vazyme, Q341‐02) was used according to the manufacturer's guidelines. The cycle threshold (CT) values were adjusted based on the β‐actin CT value, and the relative mRNA expression levels of mRNA were calculated using the 2^−ΔΔCt^ method. The primers are listed in Table S1.

### 4.12 ChIP‐qPCR

ChIP‐qPCR was conducted according to the manufacturer's protocol (Beyotime Biotechnology ChIP assay kit, P2078). Briefly, formaldehyde (37%) was used to crosslink the cells to fix the binding of protein and DNA, followed by the addition of cell expansion buffer to lyse the cells, and then micrococcal nuclease (MNase) was used for chromatin fragmentation. Next, 10% of the samples were taken as input control, and the rest of the samples were immunoprecipitated by adding specific antibodies and protein A/G magnetic beads. After washing to remove impurities, elution buffer was added to uncrosslink and release the DNA. Then the DNA was purified using PCR purification kits. Finally, the binding of the target proteins to the specific DNA sequences was examined by qPCR for quantitative analysis. The primers used are listed in Table S2.

### 4.13 ROS and lipid peroxidation assay

Cells were cultured in triplicate in six‐well plates 24 h prior to treatment, and then pre‐treated or not pre‐treated with drugs for 24 h. After removal of complete culture medium, serum‐free medium was used to prepare a final concentration of 10 µM ROS dye (DCFH‐DA, Beyotime, S0033M) or a final concentration of 5 µM lipid peroxidation dye (BODIPY 581/591 C11, Sigma‐Aldrich, D3861) for incubation at 37°C for 1 h. To remove excess dye, cells were washed with PBS more than twice, then digested with trypsin, and the digestion was terminated with complete culture medium to obtain the cells for testing. After washing the cells three times with PBS, they were resuspended in PBS containing 5% FBS. Subsequently, ROS and lipid peroxidation levels were analysed by flow cytometry using a flow cytometer (BD, Fortessa) with the fluorescein isothiocyanate (FITC) channel. All samples were analysed with FlowJo software.

### 4.14 Lipid peroxidation and ROS detection in tumour tissue

The tumour tissue from nude mice was minced into a pulp and digested using PBS containing 2 mg/mL collagenase IV,  0.5 mg/mL collagenase IV and DNase as the tissue digestion solution. The digestion was carried out in a 37°C water bath for 30 min until no adherent tissue chunks were present. The resulting solution was filtered and centrifuged using a 30‐µm filter, the supernatant was discarded, and the pellet was resuspended in PBS to obtain a single‐cell suspension. A suitable amount of the single‐cell suspension was combined with lipid peroxidation and ROS probes to achieve final concentrations of 10 µM DCFH‐DA and 5 µM BODIPY 581/591 C11. The mixture was then incubated on a 37°C constant temperature shaker for 1 h. After incubation, the cells were washed with PBS, resuspended in PBS containing 5% FBS, and the levels of ROS and lipid peroxidation in the tumour tissue were analysed using a flow cytometer (BD, Fortessa). All samples analysed with FlowJo software.

### 4.15 EdU proliferation assay flow cytometry kits (Cy5‐labelled)

The AGS, HGC‐27 and MFC cells in logarithmic growth phase were seeded into the wells of a six‐well plate. They were then incubated in a 37°C, 5% CO_2_ cell culture incubator for 24 h to allow the cells to recover and resume normal growth. After this, drug treatment, such as nutlin3a, was administered for 48 h. A 10 mM EdU stock solution was diluted to a 20 µM 2 × EdU working solution using complete culture medium. The cells were then incubated at 37°C in a CO_2_ incubator for 2 h. After trypsin digestion, the cells were precipitated. Subsequently, they were fixed in 4% PFA for 15 min and permeabilised with  0.3% Triton‐100 in PBS for 10 min at RT. Upon completion of permeabilisation, the cells were labelled with the fluorescent probe Cy5 via click chemistry, followed by resuspension in PBS containing 5% FBS for analysis using a flow cytometer. The Cy5 Azide has a maximum excitation wavelength of 646 nm and a maximum emission wavelength of 662 nm, with detection in the PE‐Cy5 channel.

### 4.16 Cell colony formation assay

Collect cells in the exponential growth phase and distribute them into a six‐well plate, grouping them according to AGS treatment into the NC group and the sgOTUD5 group. Add 1000 cells per well and set up three biological replicates. Top up each well to a final volume of 2 mL with complete growth medium, mix using a cross/eight‐shaped pattern, and then culture overnight in a 37°C, 5% CO_2_ cell culture incubator. Change the medium to complete growth medium containing 20% FBS, with a volume of 2 mL, and change the medium every 2 days. After 10–14 days, visible cell colonies should be observed by the naked eye, and at this point, they can be removed for staining. First, fix with 4% PFA for 15 min, then stain with crystal violet solution for 30 min, washing with PBS until there is no excess crystal violet at the bottom. After air drying at RT, photograph and count the number of cell colonies in each well (each colony containing more than 50 cells). Use ImageJ software to count the number of cell colonies in each well based on the obtained photos.

### 4.17 Statistical analysis

Statistical analysis was performed using GraphPad Prism 8 software. Student's *t*‐test was used to analyse the normally distributed data, while non‐parametric test was used to analyse abnormal distributed data. Log‐rank test was used for mouse survival assay. Spearman's rank test was used to analyse the correlations of IHC staining. Statistical significance was defined as a *p* value of less than  0.05. Levels of significance were indicated as *p < 0.05, **p < 0.01, ***p < 0.001, ****p < 0.0001.

## AUTHOR CONTRIBUTIONS

Junjing Zhang and Tongguan Tian played major roles in designing and doing the experiments, analysing the results and organising the figures. Xinxing Li and Kai Xu provided human GC samples and demonstrated the clinical relevance. Yao Lu, Xia Li, Xinyu Zhao, Ziyi Cui, Zhenxiang Wang, Yuefan Zhou, Yixin Xu, Hongchen Li, Yan Zhang and Yu Du helped to conduct the experiments. Junjing Zhang and Yanping Xu wrote the manuscript. Yanping Xu and Lei Lv conceived and designed the study. Yanping Xu supervised the study.

## CONFLICT OF INTEREST STATEMENT

The authors declare no conflicts of interest.

## ETHICS STATEMENT

Informed consent was obtained from all subjects, and the study was ethically approved by Ethics Committee of Tongji Hospital affiliated to Tongji University. All animal experiments were executed in accordance with the ethical obligations approved by the department of laboratory animal science of Tongji University.

## Supporting information



Supporting Information

Supporting Information

Supporting Information

## Data Availability

This study did not generate new unique reagents. Further information and requests for resources and reagents should be directed to and will be fulfilled by Yanping Xu (yanpingxu@tongji.edu.cn).
