# Supplementary material for: p53 inhibits OTUD5 transcription to promote GPX4 degradation and induce ferroptosis in gastric cancer
Source: Clin Transl Med. 2025 Mar 11;15(3):e70271. doi: 10.1002/ctm2.70271 (PMC11897053; doi:10.1002/ctm2.70271)
Supplement: Supplementary file 1 — Supporting Information [file CTM2-15-e70271-s001.docx]

**SUPPORTING INFORMATION**

**Supplemental figures**


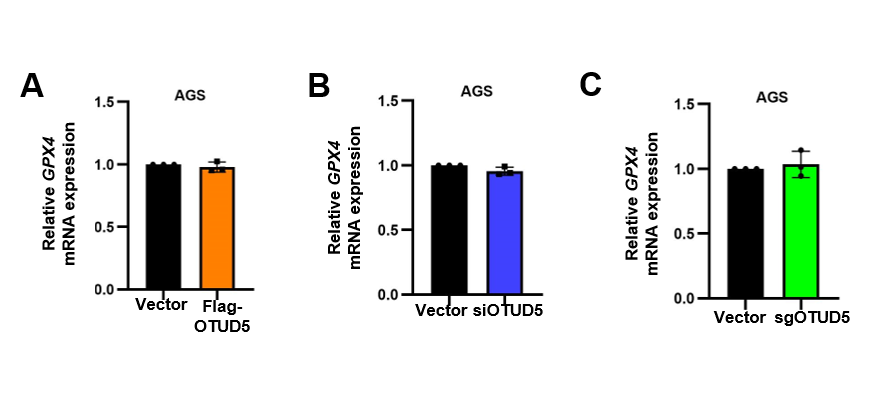


**Figure S1.**

**(A-C)** Comparison of GPX4 mRNA levels in AGS transfected with Flag-OTUD5(A) or siOTUD5(B) or sgOTUD5(C). Statistical significance was determined by Student’s t test. Data are shown as the mean ± SD (n = 3). **P < 0.01, ***P < 0.001.


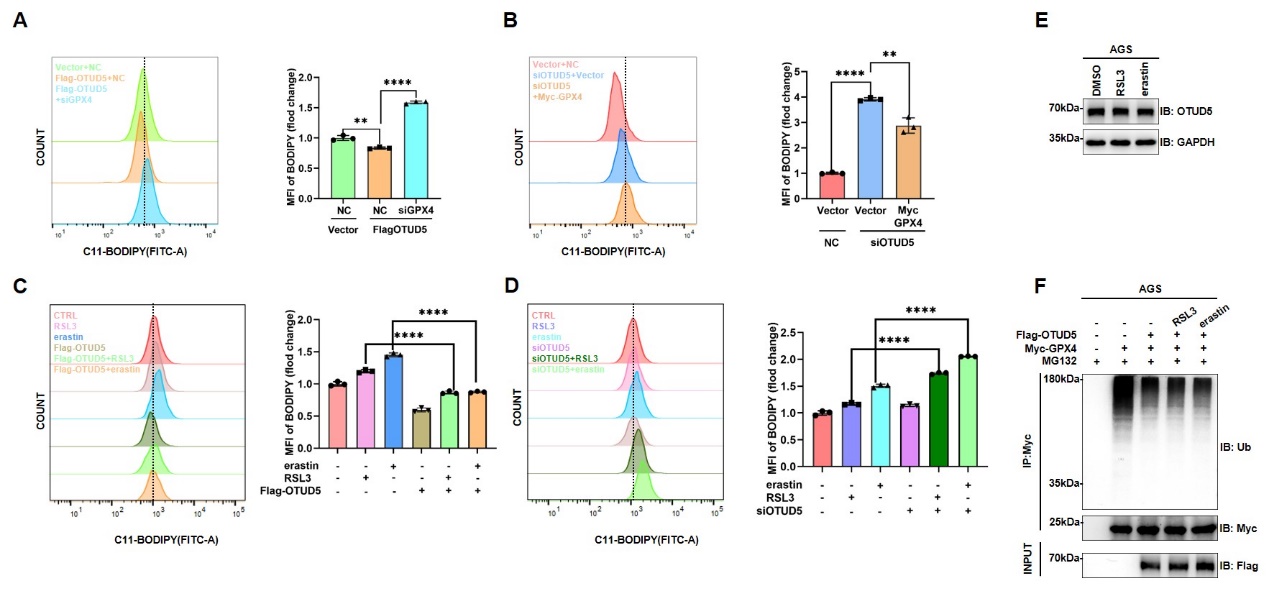


**Figure S2.**

**(A)** Flow assay of lipid peroxidation levels in AGS cells after knockdown of GPX4 followed by overexpression of Vector or Flag-OTUD5.

**(B)** Flow assay of lipid peroxidation levels in AGS cells after overexpression of Vector or GPX4 followed by knockdown OTUD5.

**(C)** Lipid peroxidation levels were determined in AGS cells transfected with Vector or Flag-OTUD5 and treated with erastin (2 μM) or RSL3 (2 μM) for 24h.

**(D)** Lipid peroxidation levels were determined in AGS cells transfected with NC or siOTUD5 and treated with erastin (2 μM) or RSL3 (2 μM) for 24h.

**(E)** Western blot analysis of OTUD5 levels in AGS treated with erastin (2 μM) or RSL3 (2 μM) as indicated.

**(F)** Western blot analysis of GPX4 ubiquitination derived from AGS cells transfected with indicated plasmids for 24h and treated with erastin (2 μM) or RSL3 (2 μM) for another 24h. GPX4 proteins were immunoprecipitated with Myc beads.


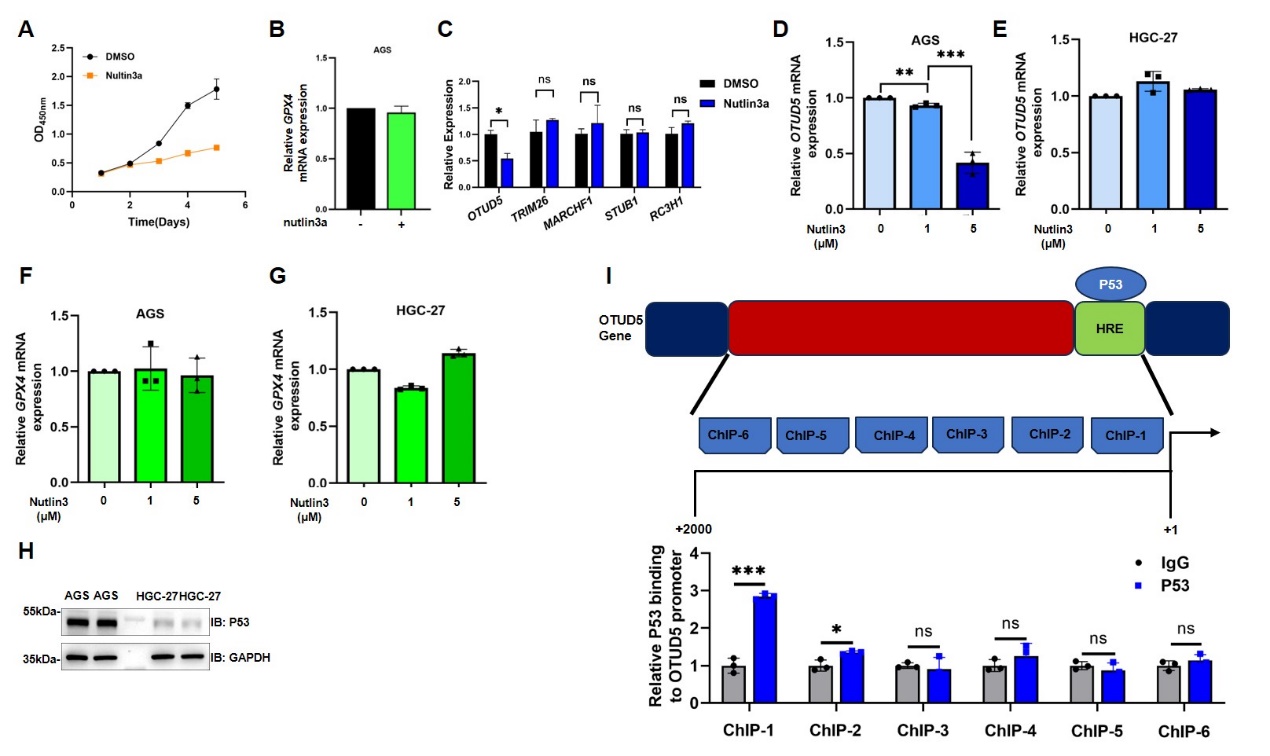


**Figure S3.**

1. CCK-8 assay of cell viability in AGS treated with nutlin3a for 5 Days.

**(B-C)** Comparison of OTUD5/TRIM26/MARCHF1/STUB1/RC3H1/GPX4 mRNA levels in AGS treated with 5 μM nutlin3a for 24h as indicated. Statistical significance was determined by Student’s t test.Data are shown as the mean±SD (n = 3). **P < 0.01, ***P < 0.001.

**(D-E)** Comparison of OTUD5 mRNA levels in AGS and HGC-27 treated with 0, 1, 5 μM nutlin3a for 24h as indicated. Statistical significance was determined by Student’s t test. Data are shown as the mean ± SD (n = 3). **P < 0.01, ***P < 0.001.

**(F-G)** Comparison of GPX4 mRNA levels in AGS and HGC-27 treated with 0, 1, 5 μM nutlin3a for 24h as indicated. Statistical significance was determined by Student’s t test. Data are shown as the mean ± SD (n = 3). **P < 0.01, ***P < 0.001.

**(H)** Western blot of p53 in AGS and HGC-27 cells is shown.

**(I)** Chromatin immunoprecipitation (ChIP) analysis of p53 binding to the OTUD5 promoter in AGS cells using anti-P53 monoclonal antibody (mAb) was conducted.
