# Supplementary material for: p53 inhibits OTUD5 transcription to promote GPX4 degradation and induce ferroptosis in gastric cancer
Source: Clin Transl Med. 2025 Mar 11;15(3):e70271. doi: 10.1002/ctm2.70271 (PMC11897053; doi:10.1002/ctm2.70271)
Supplement: Supplementary file 2 — Supporting Information [file CTM2-15-e70271-s003.docx]

**Supplemental tables**

**Table S1. qPCR Sequences**

| Name | | Target sequance（5’→3’） | |
| --- | --- | --- | --- |
| Human-OTUD5-F | GGTTGTGCGAAAGCATTGCAT | |  |
| Human-OTUD5-R | ACCTCCACAGGACGGTTGT | |  |
| Human-GPX4-F | TCGGCCGCCTTTGCC | |  |
| Human-GPX4-R | ACTTCGGTCTTGCCTCACTG | |  |
| Human-GAPDH-F | ATCAATGGAAATCCCATCACCA | |  |
| Human-GAPDH-R | GACTCCACGACGTACTCAGCG | |  |
| Human-RC3H1-F | ATGTGCCTGAATAAACTCCACC | |  |
| Human-RC3H1-R | CTTCAACCCCACTACACAAAGT | |  |
| Human-TRIM26-F | TGCACTACTACTGTGAGGACG | |  |
| Human-TRIM26-R | TCCTTAGGGTACTCAGGTGGT | |  |
| Human-MARCHF1-F | CCAAGCTCAAACCCCTCCG | |  |
| Human-MARCHF1-R | AGACCAAACCACACAGGTGAT | |  |
| Human-STUB1-F | AGCAGGGCAATCGTCTGTTC | |  |
| Human-STUB1-R | CAAGGCCCGGTTGGTGTAATA | |  |
| Human-p53-F | CAGCACATGACGGAGGTTGT | |  |
| Human-p53-R | TCATCCAAATACTCCACACGC | |  |

**Table S2. OTUD5 ChIP-qPCR Sequences**

| Name | Target sequance（5’→3’） |
| --- | --- |
| Human-ChIP-OTUD5-F1 | TGCGTTCCAAACCTTGTGCT |
| Human-ChIP-OTUD5-R1 | CCCACCTATCCGTAAGGCCA |
| Human-ChIP-OTUD5-F2 | CCTGTAGTTTGCAGCCTTGTT |
| Human-ChIP-OTUD5-R2 | CAAAACCAACCAGAAGCAGCAG |
| Human-ChIP-OTUD5-F3 | ATCGCTGATAACACCAGGACT |
| Human-ChIP-OTUD5-R3 | CTAGAAGGCAAAAGTGTGCAA |
| Human-ChIP-OTUD5-F4 | TCCTCTAGCCCTTTGTCAGGT |
| Human-ChIP-OTUD5-R4 | GTGGCTCTACCTCAAGGAGTT |
| Human-ChIP-OTUD5-F5 | TGCAAGGTCAACAGAAGCCA |
| Human-ChIP-OTUD5-R5 | ACTTCCAAAAGGGTCCTAGGTG |
| Human-ChIP-OTUD5-F6 | GCGTCCTCTCTGTCAATGATCT |
| Human-ChIP-OTUD5-R6 | TCAGCAACAGGGAAAACCTG |
