# Supplementary figures and images for: p53 inhibits OTUD5 transcription to promote GPX4 degradation and induce ferroptosis in gastric cancer
Source: Clin Transl Med. 2025 Mar 11;15(3):e70271. doi: 10.1002/ctm2.70271 (PMC11897053; doi:10.1002/ctm2.70271)

**Fig.S1**

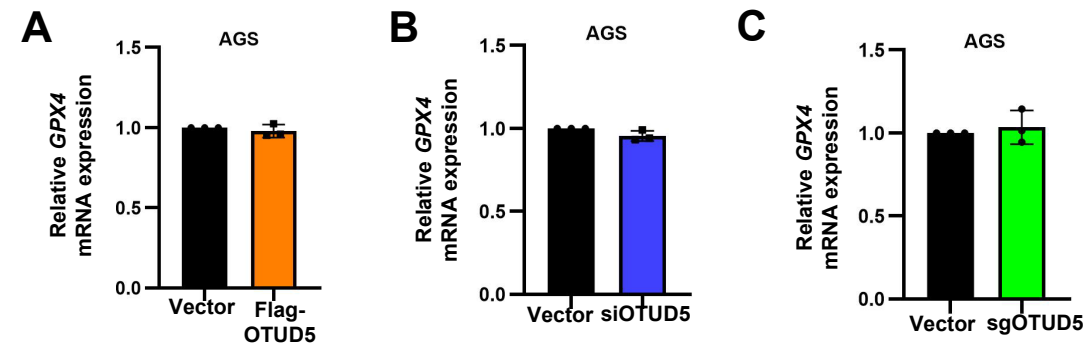

Fig.S2

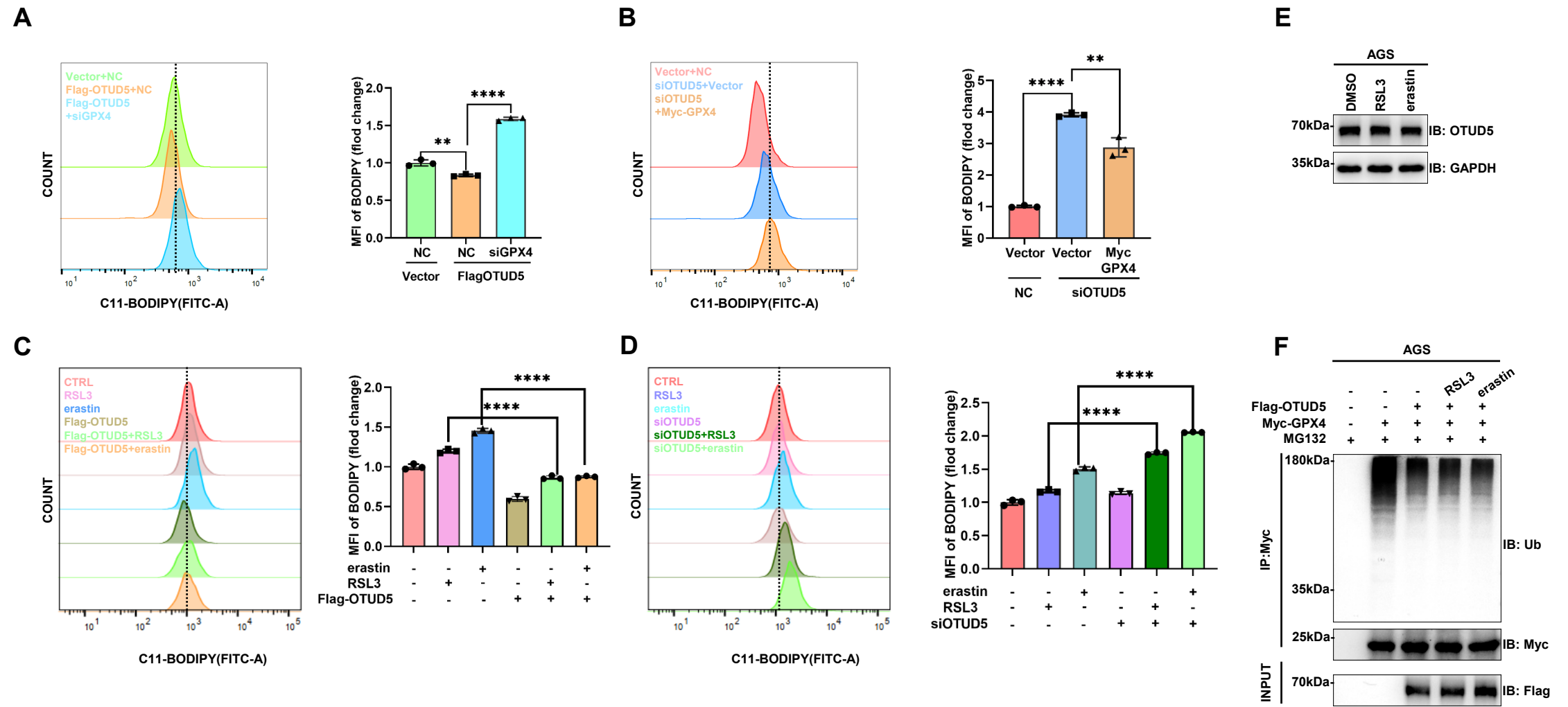

Fig.S3

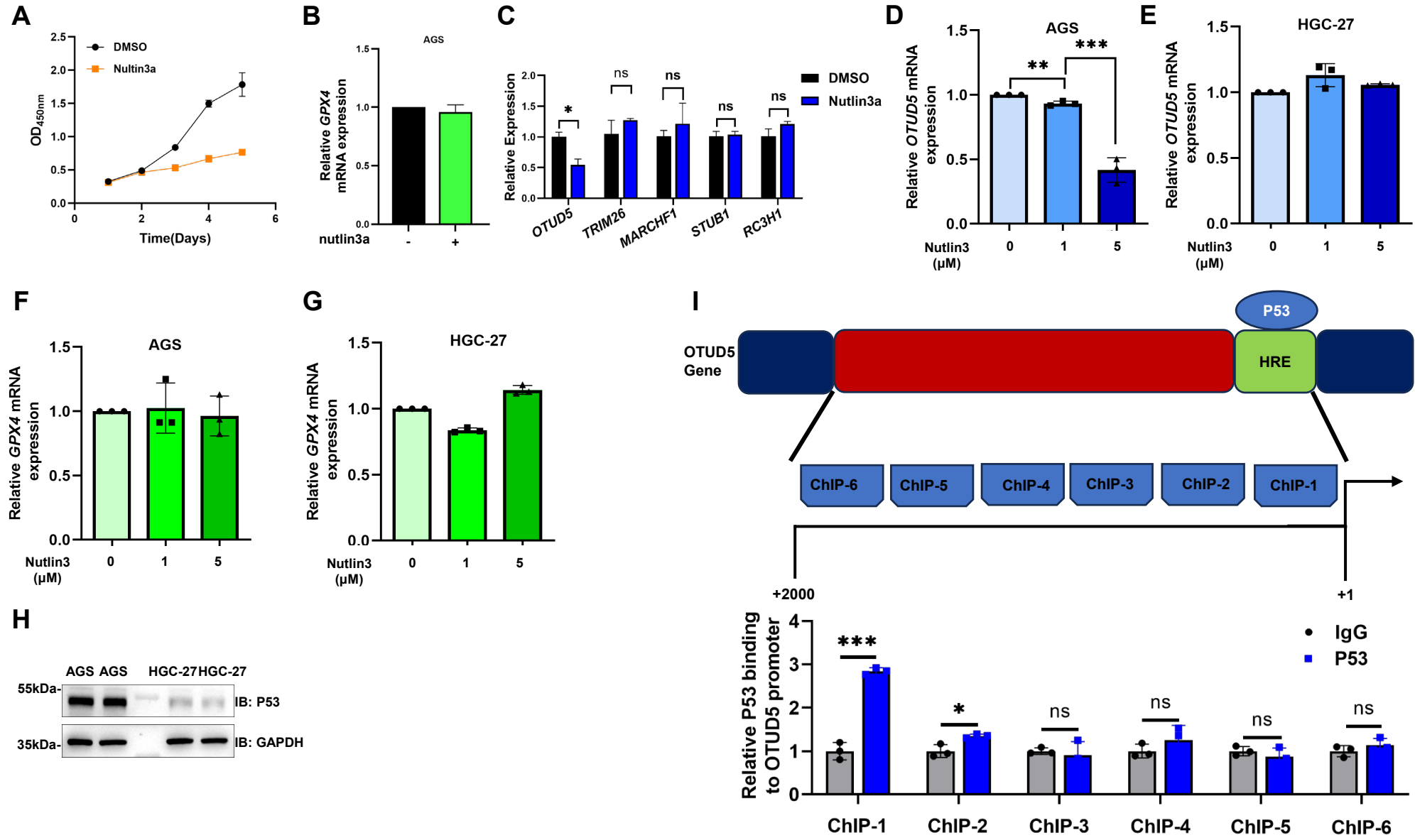

Supplement: Supplementary file 3 — Supporting Information [file CTM2-15-e70271-s002.pdf]
